# Supplementary material for: Structural insights into ATP hydrolysis by the MoxR ATPase RavA and the LdcI-RavA cage-like complex
Source: Commun Biol. 2020 Jan 28;3:46. doi: 10.1038/s42003-020-0772-0 (PMC6987120; doi:10.1038/s42003-020-0772-0)
Supplement: Supplementary file 1 — Descriptions of additional supplementary files [file 42003_2020_772_MOESM1_ESM.pdf]

Description of Additional Supplementary Files

Supplementary Data 1.
Source data for Figure 6c: Bio-Layer Interferometry (BLI) data.

Source data for Figure 6a & 6b: ATPase activity measurements.
