# Supplementary material for: Structural insights into ATP hydrolysis by the MoxR ATPase RavA and the LdcI-RavA cage-like complex
Source: Commun Biol. 2020 Jan 28;3:46. doi: 10.1038/s42003-020-0772-0 (PMC6987120; doi:10.1038/s42003-020-0772-0)
Supplement: Supplementary file 3 — supplementary information [file 42003_2020_772_MOESM3_ESM.pdf]

Supplementary information

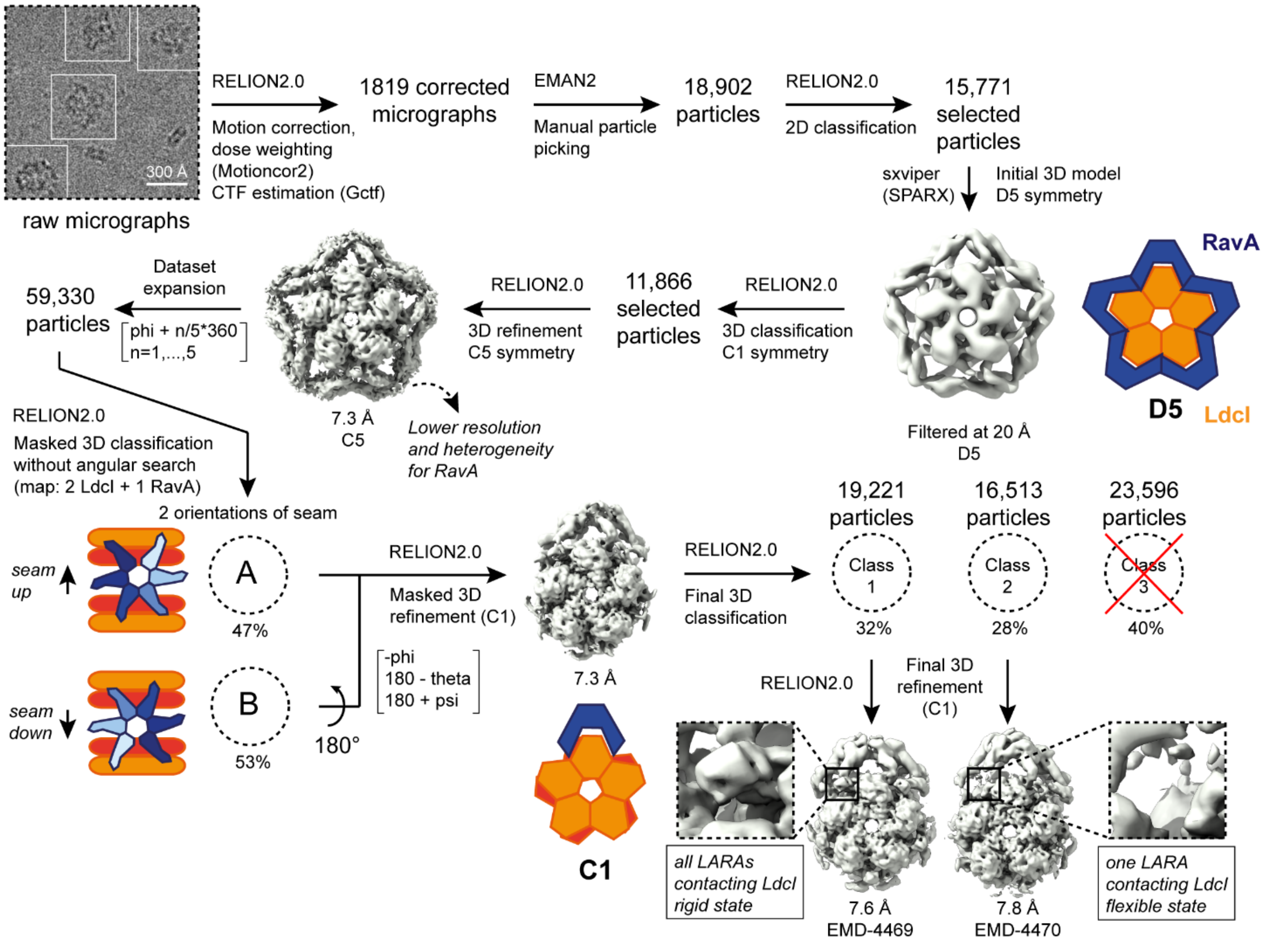

Supplementary Figure 1. Image processing pipeline of cryo-EM data of the LdcI-RavA complex supplemented with ADP. A representative micrograph is shown with picked LdcI-RavA particles, followed by subsequent classification and refinement procedures performed in this study. Software packages used at each step are indicated as well as details regarding the procedure. Schematic representations either show a top view of the LdcI-RavA cage centered on one orange-coloured LdcI decamer and five surrounding dark-blue coloured RavA hexamers, or a side view centered on one light to dark blue-coloured RavA hexamer, and two LdcI decamers with the two rings per decamer coloured light and dark orange. A summary of cryo-EM data collection, refinement and validation statistics can be found in Supplementary Table 1.

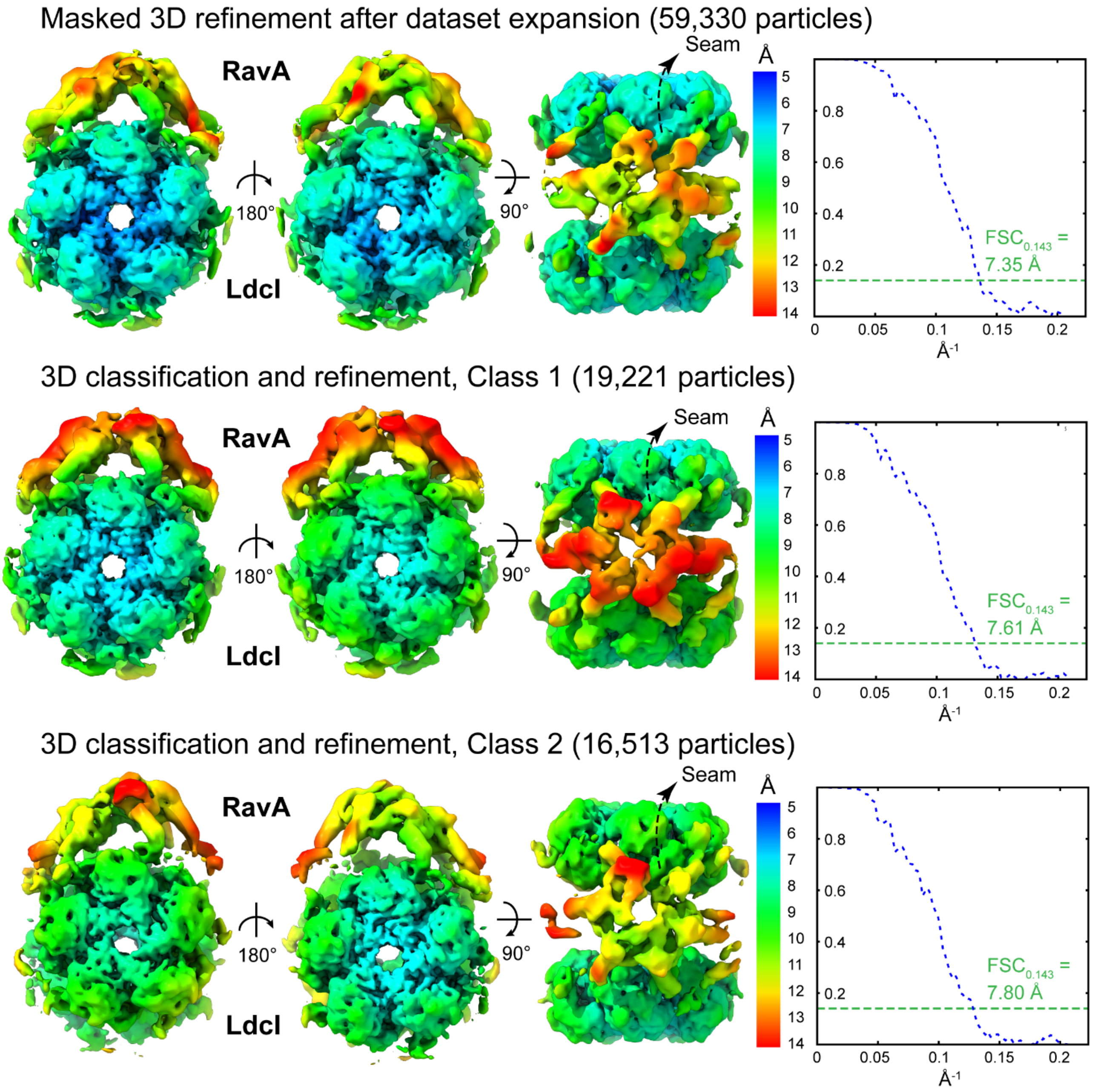

Supplementary Figure 2. Local resolution estimation in RELION2.0 for a masked 3D refinement containing all LdcI-RavA particles (upper panels), particles from LdcI-RavA Class 1 (middle panels) or particles from LdcI-RavA Class 2 (lower panels). Maps are coloured according to the local resolution. Panels on the right display gold-standard FSC curves with estimated resolution at FSC = 0.143.

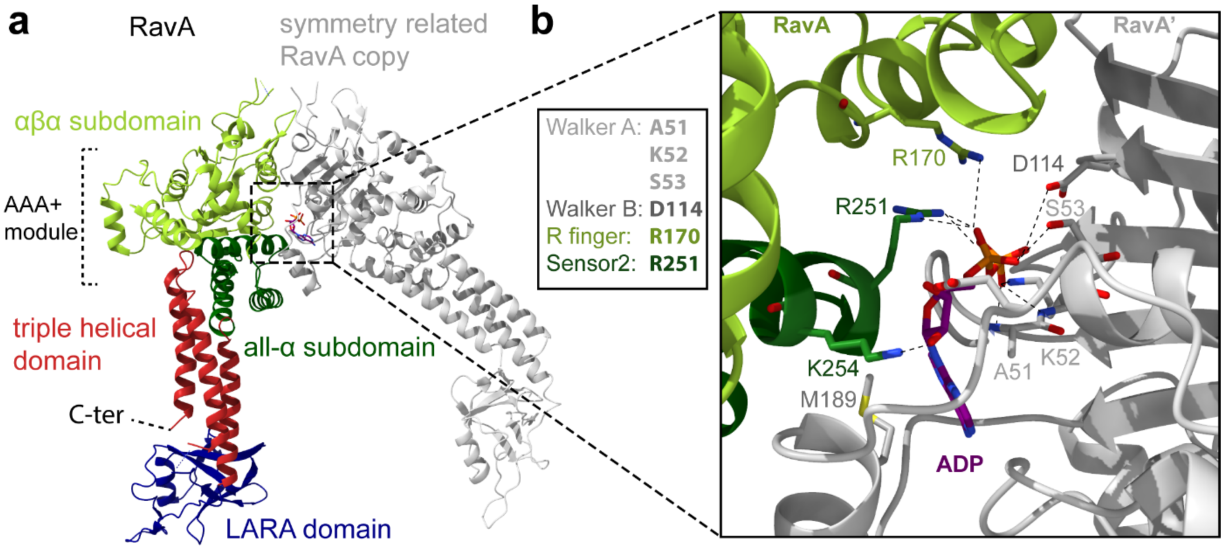

Supplementary Figure 3. (**a**): The crystal structure of *E. coli* RavA (PDB ID: 3NBX) (El Bakkouri *et al*., 2010) is shown as a cartoon. Different subdomains in the RavA monomer are annotated and coloured accordingly: AAA+ module αβα subdomain in light green, AAA+ module all-α subdomain in dark green, triple helical domain in red and LARA domain in dark blue. One symmetry related RavA copy is shown in light grey. The interface formed between the two monomers containing bound ADP is marked with a dashed square. (**b**): Zoom displaying bound ADP at the active site formed between two RavA monomers. Interacting residues are annotated and shown as sticks, polar interactions are shown as dashed lines.

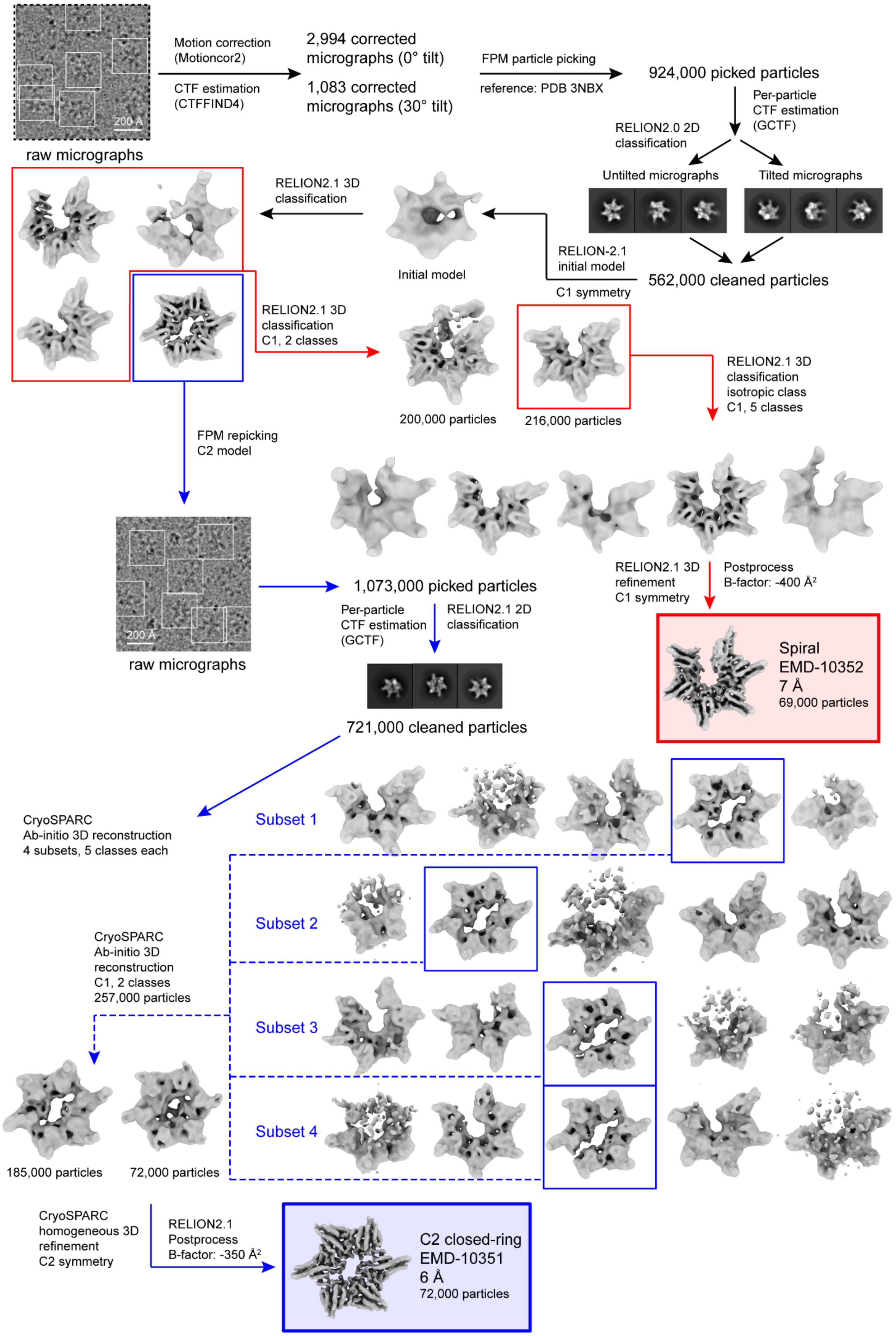

raw micrographs
200 Å
Motion correction
(Motioncor2)
CTF estimation
(CTFFIND4)
2,994 corrected
micrographs (0° tilt)
1,083 corrected
micrographs (30° tilt)
FPM particle picking
reference: PDB 3NBX
924,000 picked particles
Per-particle
CTF estimation
(GCTF)
RELION2.0 2D
classification
Untilted micrographs
Tilted micrographs
562,000 cleaned particles
RELION-2.1
initial model
C1 symmetry
Initial model
RELION2.1 3D
classification
RELION2.1 3D
classification
C1, 2 classes
200,000 particles
216,000 particles
RELION2.1 3D
classification
isotropic class
C1, 5 classes
FPM repicking
C2 model
raw micrographs
200 Å
1,073,000 picked particles
Per-particle
CTF estimation
(GCTF)
RELION2.1 2D
classification
721,000 cleaned particles
RELION2.1 3D
refinement
C1 symmetry
Postprocess
B-factor: -400 Å²
Spiral
EMD-10352
7 Å
69,000 particles
CryoSPARC
Ab-initio 3D reconstruction
4 subsets, 5 classes each
Subset 1
Subset 2
Subset 3
Subset 4
CryoSPARC
Ab-initio 3D
reconstruction
C1, 2 classes
257,000 particles
185,000 particles
72,000 particles
CryoSPARC
homogeneous 3D
refinement
C2 symmetry
RELION2.1
Postprocess
B-factor: -350 Å²
C2 closed-ring
EMD-10351
6 Å
72,000 particles

Supplementary Figure 4. Image processing pipeline of cryo-EM data of unbound RavA supplemented with ADP. A representative micrograph is shown with picked RavA particles, followed by subsequent classification and refinement procedures performed in this study. Software packages used at each step are indicated as well as details regarding the procedure. A summary of cryo-EM data collection, refinement and validation statistics can be found in Supplementary Table 1.

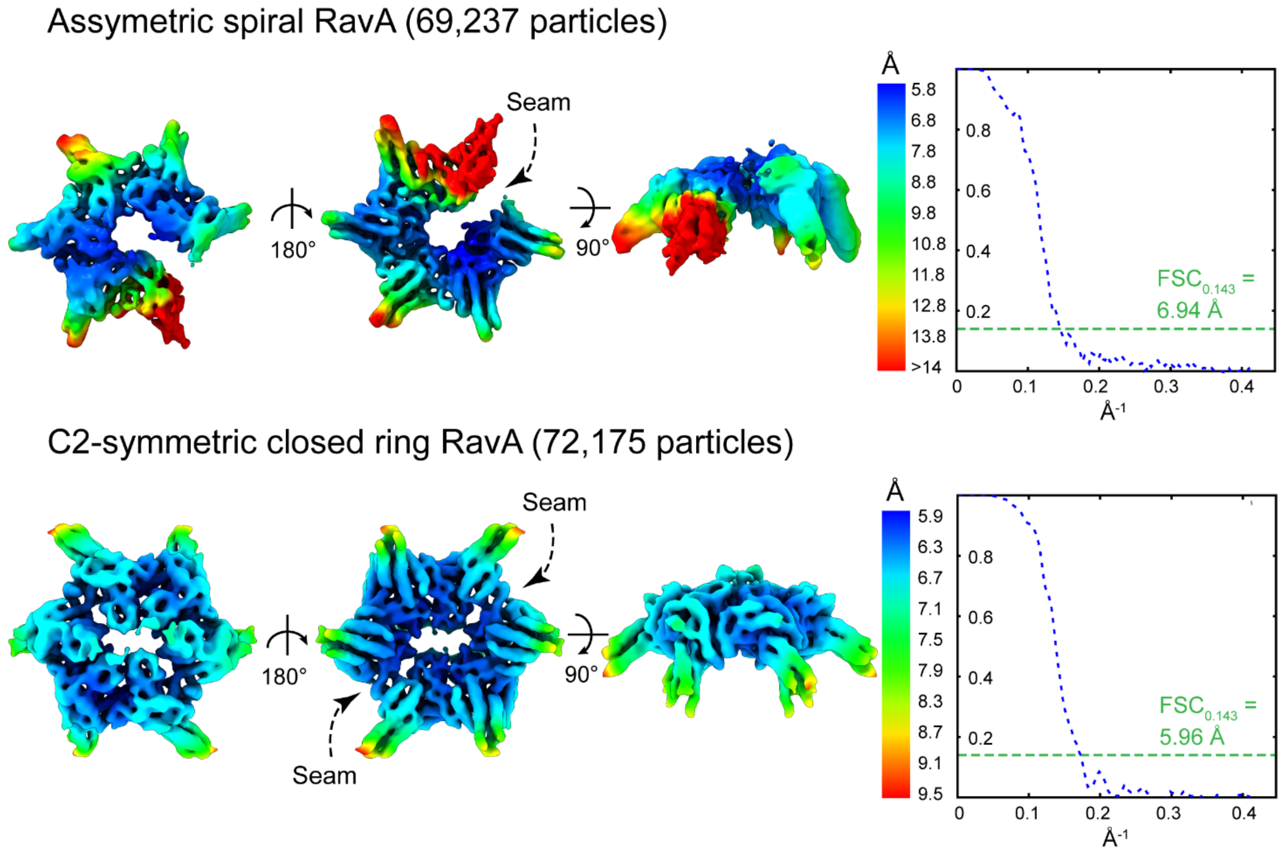

Supplementary Figure 5. Local resolution estimation in RELION3.0 for 3D refinements of spiral and C2-symmetric closed ring conformations of RavA. Maps are coloured according to the local resolution. Panels on the right display gold-standard FSC curves with estimated resolution at FSC = 0.143.

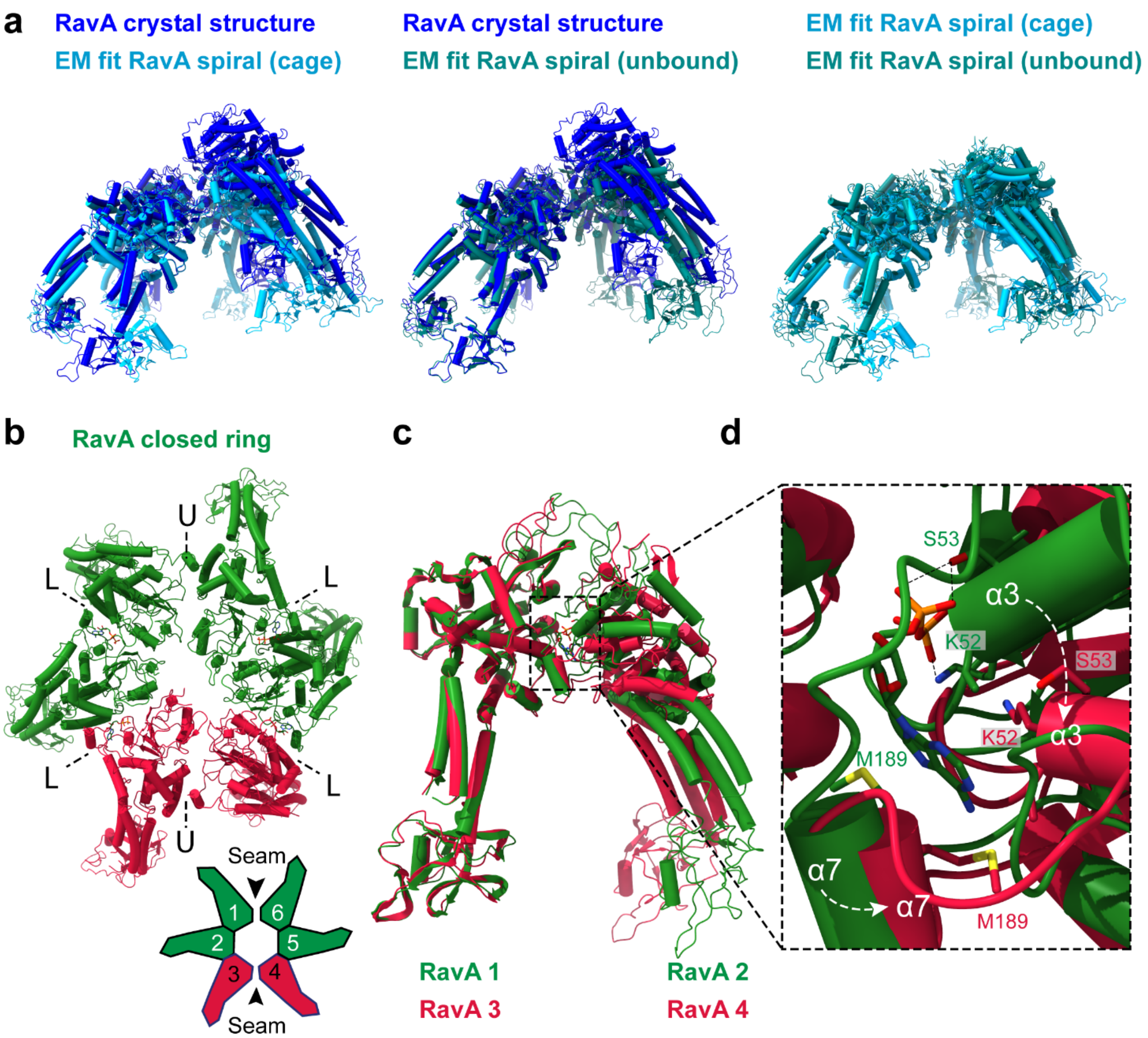

Supplementary Figure 6. (**A**) Side by side comparisons of spiral RavA hexamers extracted from the crystal structure (dark blue) and fits of RavA in the cryo-EM maps of the LdcI-RavA cage (light blue) or the spiral conformation of unbound RavA (dark cyan). Structures are displayed as cartoons. (**B**) Cartoon representation of the C2-symmetric closed ring conformation of unbound RavA. Loadable and unloadable ATP binding sites are annotated with L and U respectively. An accompanying schematic representation is shown with numbered RavA monomers. The seam positions between monomers 1-6 and 3-4 are annotated using black arrows. (**C**) Alignment of neighbouring RavA monomers 1 and 2 (green), and 3 and 4 (crimson red) extracted from the C2-symmetric closed ring conformation of unbound RavA. (**D**) Zoom of the active-site interface formed between adjacent RavA monomers 1 and 2 (green, with bound ADP) and monomers 3 and 4 (crimson red, without present nucleotide). Shifts in helices α3 and α7 are accentuated using dotted white arrows. Relevant residues are labelled and shown as sticks.

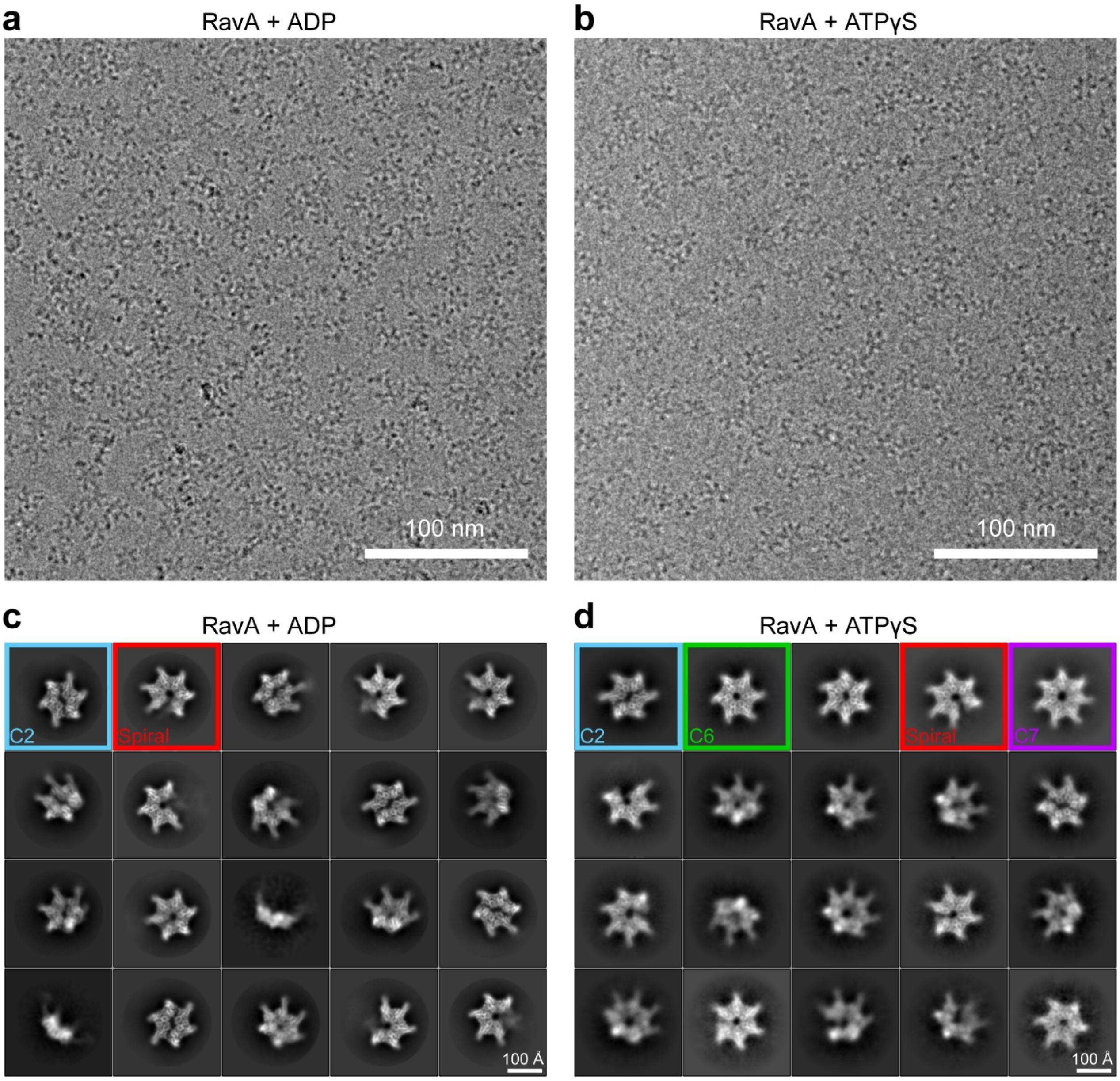

Supplementary Figure 7. (**a**) & (**b**): Micrographs of RavA supplemented with ADP (**a**) or ATPγS (**b**). (**c**) & (**d**): Representative 2D class averages displaying spiral (red squares) and C2 symmetric closed-ring (blue squares) conformations found in both the ADP (**c**) and ATPγS (**d**) datasets, while additional C6 symmetric (green square) and C7 symmetric (purple square) 2D class averages are solely found in the ATPγS dataset.

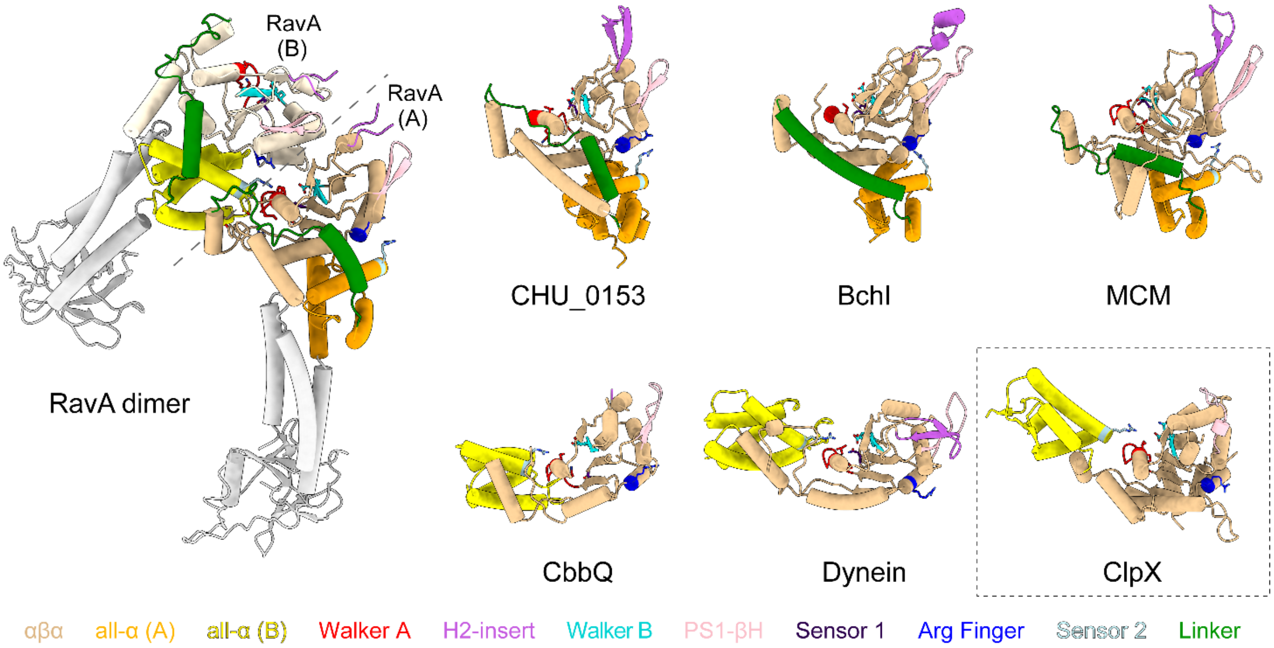

Supplementary Figure 8. Cartoon representation of ATPase domains of the Clade 7 AAA+ ATPases CHU_0153 (PDB ID 2R44), BchI (PDB ID 1G8P), MCM (PDB ID 4R7Y), CbbQ (PDB ID 5C3C), Dynein (PDB ID 4AKG, AAA3 domain) aligned to a RavA monomer (A) (PDB ID 6SZA), with ClpX (PDB ID 3HWS) shown for reference. Structural features of models are coloured as follows: αβα = light orange, all-α = orange/yellow, Walker A = red, H2-insert = purple, Walker B = cyan, PS1-βH = pink, Sensor 1 = indigo, Arginine Finger = blue, Linker = green, Sensor 2 = light blue. Where the αβα and all-α subdomains superimpose with the corresponding domains in a single RavA monomer (A), the all-α subdomain is coloured orange. Where the all-α subdomain superimposes with the all-α subdomain of the adjacent RavA monomer (B) in the RavA dimer, it is coloured yellow. RavA, CHU_0153, BchI and MCM share the reorientation of the all-α subdomain following a linker which is distinctive to clade 7 AAA+ ATPases, whereas CbbQ and Dynein align more closely to the Clade 5 ClpX, and lack the linker region. Relevant residues from the different structural features are shown as sticks.

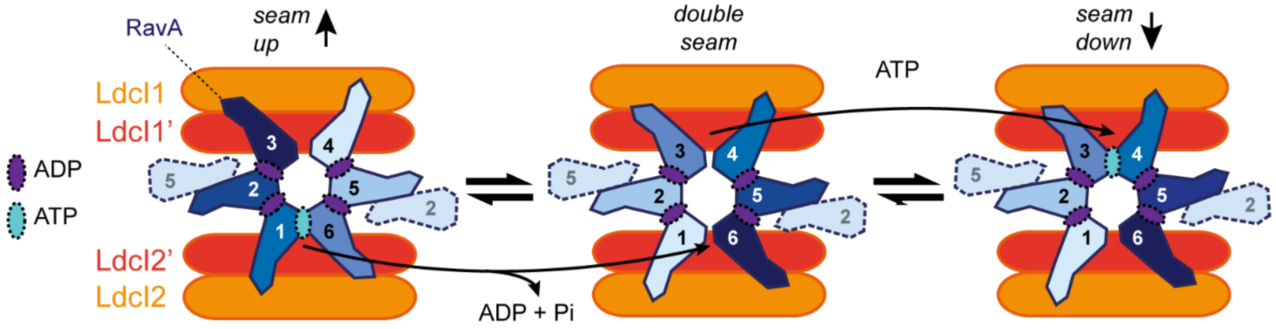

Supplementary Figure 9. Schematic representation of a possible scenario for the ATPase mechanism of LdcI-bound RavA. The two pentameric rings per LdcI decamer are coloured light and dark orange respectively, while RavA hexamers are coloured light to dark blue. ATP hydrolysis only occurs at the active sites formed between monomers 1 and 6 or 3 and 4 in the spiral RaVA hexamer. RavA legs from adjacent hexamers (dashed lines) constrain the movement of RavA monomers 2 and 5, resulting in inactive ATPase sites between monomers 1-2, 2-3, 4-5 and 5-6. As a result, the spiral RavA hexamer cycles between two equivalent states with the seam oriented towards either the upper ('seam up') or lower ('seam down') LdcI decamer during ATP hydrolysis via an intermediate 'double seam' state.

**Data collection and processing**	LdcI-RavA complex + ADP		RavA + ADP	
	Class 1<br><br>EMD-4469<br>PDB ID 6Q7L	Class 2<br><br>EMD-4470<br>PDB ID 6Q7M	C2-symmetric closed ring<br>EMD-10351<br>PDB ID 6SZA	Spiral open ring<br>EMD-10352<br>PDB ID 6SZB
Magnification	41,270	41,270	41,270	41,270
Voltage (kV)	300	300	300	300
Electron exposure ($e^{-}/Å^2$)	40	40	40	40
Defocus range (μm)	1.0 - 3.5 μm	1.0 - 3.5 μm	1.8 - 3.8 μm	1.8 - 3.8 μm
Pixel size (Å)	1.21	1.21	1.21	1.21
Symmetry imposed	C1	C1	C2	C1
Initial particle images (no.)	59,330	59,330	~1,072,000	~924,000
Final particle images (no.)	19,221	16,513	72,175	69,237
Map resolution (Å)<br>FSC threshold	7.61<br>0.143	7.80<br>0.143	5.96<br>0.143	6.94<br>0.143
Map resolution range (Å)	6.5 - 14.9	6.9 - 14.8	5.9-10.0	5.8 – 23.0
**Fitting/Refinement**				
Used Software	iMODFIT, Phenix	iMODFIT, Phenix	iMODFIT, Phenix	iMODFIT, Phenix
Initial model used (PDB code)	3NBX<br>3N75	3NBX<br>3N75	3NBX	3NBX
Model resolution (Å)<br>FSC threshold	8.8<br>0.5	9.7<br>0.5	7.1<br>0.5	8.0<br>0.5
Model resolution range (Å)	∞ - 8.8	∞ - 9.7	∞ - 7.1	∞ - 8.0
Map sharpening *B* factor ($Å^2$)	-250	-300	-350	-400
Model composition<br>Non-hydrogen atoms<br>Protein residues<br>Ligands (ADP)	 137,337<br>17,102<br>5	 137,351<br>17,104<br>5	 23,190<br>2886<br>4	 23,217<br>2886<br>5
B factors ($Å^2$)<br>Protein<br>Ligand	 371.76<br>740.63	 288.39<br>401.93	 425.58<br>224.85	 551.42<br>344.86
R.m.s. deviations<br>Bond lengths (Å)<br>Bond angles (°)	 0.003<br>0.462	 0.003<br>0.466	 0.018<br>0.91	 0.003<br>0.870
Validation<br>MolProbity score<br>Clashscore<br>Poor rotamers (%)	 1.90<br>6.17<br>0.17	 1.91<br>6.13<br>0.16	 1.76<br>5.86<br>0.92	 1.66<br>4.42<br>0.88
Ramachandran plot<br>Favored (%)<br>Allowed (%)<br>Disallowed (%)	 89.41<br>9.92<br>0.67	 89.27<br>9.99<br>0.74	 93.19<br>6.14<br>0.67	 93.19<br>6.14<br>0.67

Supplementary Table 1. Cryo-EM data collection, refinement and validation statistics

	K_D (M)	k_{on} (1/Ms)	k_{dis} (1/s)
**RavA-AVI + LdcI, pH 7.0**	4.63E-08 ± 4.28E-10	2.13E+04 ± 1.27E+02	9.87E-04 ± 6.98E-06
**RavA-AVI + ADP + LdcI, pH 7.0**	2.67E-08 ± 2.97E-10	2.47E+04 ± 1.33E+02	6.59E-04 ± 6.40E-06
**RavA-AVI + ADP + LdcI, pH 8.0**	4.42E-08 ± 4.72E-10	2.96E+04 ± 2.24E+02	1.31E-03 ± 9.86E-06
**RavA-AVI + ADP + LdcI, pH 6.5**	2.09E-08 ± 2.73E-10	2.57E+04 ± 1.42E+02	5.38E-04 ± 6.38E-06
**RavA-AVI + ADP + LdcI, pH 5.0**	2.71E-08 ± 2.42E-10	2.73E+04 ± 1.27E+02	7.40E-04 ± 5.63E-06

Supplementary Table 2. Summary of BLI measurements of the LdcI-RavA interaction at different pH values.
