# Supplementary material for: Structural insights into ATP hydrolysis by the MoxR ATPase RavA and the LdcI-RavA cage-like complex
Source: Commun Biol. 2020 Jan 28;3:46. doi: 10.1038/s42003-020-0772-0 (PMC6987120; doi:10.1038/s42003-020-0772-0)
Supplement: Supplementary file 4 — Reporting Summary [file 42003_2020_772_MOESM4_ESM.pdf]

nature research

Corresponding author(s): Irina Gutsche

Last updated by author(s): Nov 28, 2019

Reporting Summary

Nature Research wishes to improve the reproducibility of the work that we publish. This form provides structure for consistency and transparency in reporting. For further information on Nature Research policies, see Authors & Referees and the Editorial Policy Checklist.

Statistics

For all statistical analyses, confirm that the following items are present in the figure legend, table legend, main text, or Methods section.

n/a	Confirmed	
☐	☒	The exact sample size (*n*) for each experimental group/condition, given as a discrete number and unit of measurement
☐	☒	A statement on whether measurements were taken from distinct samples or whether the same sample was measured repeatedly
☒	☐	The statistical test(s) used AND whether they are one- or two-sided<br>*Only common tests should be described solely by name; describe more complex techniques in the Methods section.*
☒	☐	A description of all covariates tested
☒	☐	A description of any assumptions or corrections, such as tests of normality and adjustment for multiple comparisons
☐	☒	A full description of the statistical parameters including central tendency (e.g. means) or other basic estimates (e.g. regression coefficient) AND variation (e.g. standard deviation) or associated estimates of uncertainty (e.g. confidence intervals)
☒	☐	For null hypothesis testing, the test statistic (e.g. *F*, *t*, *r*) with confidence intervals, effect sizes, degrees of freedom and *P* value noted<br>*Give P values as exact values whenever suitable.*
☒	☐	For Bayesian analysis, information on the choice of priors and Markov chain Monte Carlo settings
☒	☐	For hierarchical and complex designs, identification of the appropriate level for tests and full reporting of outcomes
☒	☐	Estimates of effect sizes (e.g. Cohen's *d*, Pearson's *r*), indicating how they were calculated

Our web collection on statistics for biologists contains articles on many of the points above.

Software and code

Policy information about availability of computer code

Data collection	Latitude, EPU
Data analysis	EMAN2<br>sxviper (SPARX)<br>Relion2.0<br>Relion2.1<br>Relion3<br>MotionCor2<br>CTFfind4<br>Gautomatch<br>FPM<br>GCTF<br>ResMap<br>iMODFIT<br>Phenix<br>Coot<br>Pymol<br>Chimera<br>ChimeraX<br>FortéBio Data Analysis 9.0 Software

For manuscripts utilizing custom algorithms or software that are central to the research but not yet described in published literature, software must be made available to editors/reviewers. We strongly encourage code deposition in a community repository (e.g. GitHub). See the Nature Research guidelines for submitting code & software for further information.

Data

Policy information about availability of data

All manuscripts must include a data availability statement. This statement should provide the following information, where applicable:

- Accession codes, unique identifiers, or web links for publicly available datasets
- A list of figures that have associated raw data
- A description of any restrictions on data availability

- Cryo-EM maps of the two classes of the LdcI-RavA complex in ADP-bound state, and the spiral and C2-symmetric closed ring conformations of free RavA in the presence of ADP, together with their corresponding fitted atomic structures have been submitted to the EMDB and PDB with accession codes EMD-4469 and PDB-6Q7L for LdcI-RavA Class 1, EMD-4470 and PDB-6Q7M for LdcI-RavA Class 2, EMD-10351 and PDB-6SZA for the C2-symmetric closed ring RavA conformation, and EMD-10352 and PDB-6SZB for the spiral RavA conformation.

- Figures with associated raw data (in Source file): Figure 6

- Data is available upon request

Field-specific reporting

Please select the one below that is the best fit for your research. If you are not sure, read the appropriate sections before making your selection.

☒ Life sciences ☐ Behavioural & social sciences ☐ Ecological, evolutionary & environmental sciences

For a reference copy of the document with all sections, see nature.com/documents/nr-reporting-summary-flat.pdf

Life sciences study design

All studies must disclose on these points even when the disclosure is negative.

Sample size	For cryoEM, the number of particles used for each of the EM reconstructions has been stated in the methods section.
Data exclusions	For cryoEM, particles were sorted and selected as described in the methods section.
Replication	ATPase activity measurements were performed in triplicate (technical replicates).
Randomization	For cryoEM, extracted particles were randomly assigned to two separate groups to calculate half-maps and gold-standard FSC.
Blinding	N/A

Reporting for specific materials, systems and methods

We require information from authors about some types of materials, experimental systems and methods used in many studies. Here, indicate whether each material, system or method listed is relevant to your study. If you are not sure if a list item applies to your research, read the appropriate section before selecting a response.

Materials & experimental systems

n/a	Involved in the study
☒	☐ Antibodies
☒	☐ Eukaryotic cell lines
☒	☐ Palaeontology
☒	☐ Animals and other organisms
☒	☐ Human research participants
☒	☐ Clinical data

Methods

n/a	Involved in the study
☒	☐ ChIP-seq
☒	☐ Flow cytometry
☒	☐ MRI-based neuroimaging
